# Perspectives of older adults on co-management of low back pain by doctors of chiropractic and family medicine physicians: a focus group study

**DOI:** 10.1186/1472-6882-13-225

**Published:** 2013-09-16

**Authors:** Kevin J Lyons, Stacie A Salsbury, Maria A Hondras, Mark E Jones, Andrew A Andresen, Christine M Goertz

**Affiliations:** 1 Office of Institutional Research, Thomas Jefferson University, Philadelphia, PA, USA; 2 Palmer Center for Chiropractic Research, Palmer College of Chiropractic, Davenport, IA, USA; 3 Institute of Sports Science and Clinical Biomechanics, University of Southern Denmark, Odense, Denmark; 4 Genesis Quad Cities Family Medicine Residency Program, Davenport, IA, USA

**Keywords:** Older adults, Low back pain, Health services for the aged, Interprofessional relations, Chiropractic, Family medicine, Musculoskeletal disorders, Complementary/alternative medicine, Patient preferences, Qualitative research

## Abstract

**Background:**

While older adults may seek care for low back pain (LBP) from both medical doctors (MDs) and doctors of chiropractic (DCs), co-management between these providers is uncommon. The purposes of this study were to describe the preferences of older adults for LBP co-management by MDs and DCs and to identify their concerns for receiving care under such a treatment model.

**Methods:**

We conducted 10 focus groups with 48 older adults who received LBP care in the past year. Interviews explored participants’ care seeking experiences, co-management preferences, and perceived challenges to successful implementation of a MD-DC co-management model. We analyzed the qualitative data using thematic content analysis.

**Results:**

Older adults considered LBP co-management by MDs and DCs a positive approach as the professions have complementary strengths. Participants wanted providers who worked in a co-management model to talk openly and honestly about LBP, offer clear and consistent recommendations about treatment, and provide individualized care. Facilitators of MD-DC co-management included collegial relationships between providers, arrangements between doctors to support interdisciplinary referral, computer systems that allowed exchange of health information between clinics, and practice settings where providers worked in one location. Perceived barriers to the co-management of LBP included the financial costs associated with receiving care from multiple providers concurrently, duplication of tests or imaging, scheduling and transportation problems, and potential side effects of medication and chiropractic care. A few participants expressed concern that some providers would not support a patient-preferred co-managed care model.

**Conclusions:**

Older adults are interested in receiving LBP treatment co-managed by MDs and DCs. Older adults considered patient-centered communication, collegial interdisciplinary interactions between these providers, and administrative supports such as scheduling systems and health record sharing as key components for successful LBP co-management.

## Background

Low back pain (LBP) is a leading cause of disability and disease burden [1,2]. People age 65 years and older report a 25% monthly LBP prevalence rate [3,4] with recurrent or debilitating LBP common in older populations [4-6]. Chronic LBP is linked to difficulties with activities of daily living (ADLs) [7,8], depression [4,7,9,10], sleep problems [7,9], and decreased performance on physical function [7,11] and neuropsychological tests [11]. An estimated 2.3% of annual physician visits in the U.S. are for LBP [3]. Persons with LBP and other spine conditions have increased healthcare expenditures for medications, spinal imaging, injections and surgery [3,4,9,12-14]. Medicare charges among older adults with back disorders have demonstrated significant increases for patient visits, imaging and spinal injections [12,15] without translation to better health outcomes for LBP patients [13-15].

Patients with unresolved pain may pair conventional healthcare with complementary and alternative medicine (CAM) [16-19]. Chiropractic is among the most widely used CAM therapies [16,17,20,21], including by older adults [22-26]. Patients who use medical care and chiropractic together believe the combination helps their condition more than either alone [21]. And yet, co-occurring medical and chiropractic care is uncommon among older patients, ranging from 5-11% [23] to 33% [27]. Medical doctors (MDs) and doctors of chiropractic (DCs) make few formal referrals to each other and rarely share health records, suggesting little care coordination between these providers [28-30]. While patients of all ages might benefit from improved co-management of their pain and other medical conditions [31-33], interprofessional collaboration between healthcare providers is particularly important for older adults due to the high rates of chronic disease, multimorbidity and disability [32,34-36], polypharmacy [37,38], and healthcare received from multiple providers [39] in this patient population.

The purpose of this focus group study was to explore the perspectives of older adults toward LBP collaborative care by MDs and DCs. Researchers have conducted focus groups to understand patients’ LBP experiences [40,41] and primary care preferences [42,43] and to design interventions for musculoskeletal disease [44-47]. However, researchers have not explored the patient perspective on LBP collaborative care. In this paper, we describe older adults’ LBP healthcare seeking experiences, expectations for collaborative care provided by family medicine MDs and DCs, and insights into implementation barriers and facilitators. We incorporated the recommendations from these focus groups into the design of an interdisciplinary model and training plan for DCs and MDs providing co-managed LBP care to older people that was subsequently tested in a pragmatic randomized controlled trial [48].

## Methods

Genesis Health System and Palmer College of Chiropractic institutional review boards provided ethics approval for this study. Focus group methodology allowed the researchers to gather a diverse range of perceptions of older adults who shared the experience of LBP through structured, moderated group discussions [49]. As we could identify no previous studies assessing patient preferences for LBP co-management by MDs and DCs, we could not anticipate which topics might be most important for older adults with this condition. We selected focus groups as a method that would allow for a dynamic data collection process in which group members might immediately discuss new topics introduced by fellow participants while statements offered by one individual might trigger the recollections of other members. We modified standard focus group techniques for use with older adults by using smaller groups and shorter time periods for data collection [50].

We recruited participants by letter from patient lists at a family medicine clinic and chiropractic academic health center and through flyers at two senior centers (SC) and three senior housing (SH) sites. Community-dwelling adults who were 65 years or older and self-reported LBP in the past year were eligible. Participants read and spoke English, heard well enough to join a discussion, and were willing to talk about LBP in a group. Participants provided written consent and completed a demographic survey. Sessions were held in conference rooms and lasted about one hour. Participants received a small gift and snacks for their contributions, but received no monetary compensation.

Table 1 provides the key focus group questions. An interdisciplinary steering committee developed the interview based on a literature review, discussions, and a theory of integrative medicine [51]. The lead author (KJL) facilitated most sessions while assistant moderators (SAS, MAH) documented the interview process in fieldnotes and asked follow-up questions. During the introduction to each session, the moderators instructed participants on the aims and methods of focus groups. The moderators emphasized that participants need not agree with one another, that diverse perspectives on LBP care were welcomed, and that the goal of the discussion was not to achieve consensus on the topics under discussion, but, rather, to generate new information based on the participants’ own experiences. The moderating team guided the interviews back to the main topic of discussion when irrelevant subjects arose or when a single group member dominated the conversation. The lead moderators also took care to ask the group if they had similar experiences, probed for differing opinions and invited quieter group members to offer their own perspectives to the conversation. Overall, the groups listened intently to the discussions, did not appear hesitant to discuss conflicting opinions, and offered supportive comments to participants who expressed emotional concerns. The moderating team presented an oral overview of key themes discussed at the end of the session to confirm initial session findings with participants [52]. Participants then added to or clarified points of the discussion. The moderating team debriefed after each session to identify key topics and format changes to elicit feedback from subsequent groups on emerging themes. Focus group sessions continued until thematic saturation was reached [52,53]. A transcriptionist transcribed the digitally-recorded sessions verbatim that an assistant moderator had compared to the recordings to establish accuracy [53].

**Table 1:** Focus group questions

LBP healthcare seeking	Which healthcare providers have you seen for your back condition?
	What do you expect a medical doctor to do for your back condition?
	What do you expect a doctor of chiropractic to do for your back condition?
Referral experiences	Has your medical doctor or doctor of chiropractic ever recommended that you see another health care provider for treatment for your back?
	Have you ever asked your medical doctor/doctor of chiropractic to refer you to another health care provider for your back problems?
Co-management experiences	What made/might make you decide to seek treatment from more than one health care provider for your back pain?
	Would you mention it to your medical doctor/doctor of chiropractic if you were also seeing another health care provider for your back problem? Why or why not?
	Do you think having a medical doctor and a doctor of chiropractic treating your back condition as a team would be helpful? Why or why not?
Co-management barriers and facilitators	What concerns would you have about seeing a both medical doctor and a doctor of chiropractic for this condition?
	What could a medical doctor and a doctor of chiropractic do to make it easier for you to see them both for your back problem?
	What would you do if a medical doctor and a doctor of chiropractic told you to do different treatments for your back pain?

Transcriptions were analyzed using content analysis [54]. The lead author (KJL), a social scientist with expertise in interprofessional collaboration, summarized the themes for each focus group and the overall set of groups [49]. The assistant moderators are a gerontological nurse with experience in interdisciplinary teams and qualitative methods (SAS) and a doctor of chiropractic with expertise in LBP in older adults (MAH). The assistant moderators completed a peer review process to affirm the dependability of the initial coding by reading the transcripts and group summaries independently, confirming and expanding the thematic codebook, and classifying themes into discrete domains [53,54]. We organized our analysis at the group level, rather than at the level of the individual. The analytic team organized the themes into data tables and identified which groups discussed each of the themes to identify similarities and differences in the topics generated in discussion across the four different settings from which we recruited participants. Data tables include a checkmark whenever a group discussed a topic. In some cases, many participants may have discussed a topic, while in others only one participant may have introduced an idea. In our results, we use the terms ‘most’ or ‘many’ when the majority of participants or the focus groups discussed a particular theme, ‘some’ when half of the groups identified a topic, and ‘few’ when themes were discussed by one or two participants in less than half of the groups.

## Results

### Participant and group characteristics

We conducted 10 focus groups between May 2010 and November 2011, with sessions composed of 2 to 10 participants. The sample included 48 participants (10 males and 38 females) whose mean age (SD) was 75.2 (8.0) years. Table 2 presents participant characteristics. Five groups were composed of mixed genders, while one group of family medicine patients, one senior housing group, and two senior center groups had female participants only and one chiropractic patient group had male participants only. Participants in the three senior housing groups differed from the other groups in that few owned their own cars for transportation. The chiropractic patient groups more often spoke of their personal histories of receiving chiropractic care from DCs at this particular clinic compared to members of the other focus groups. Many participants in the senior housing and senior center groups were acquaintances of one another, while the participants in the patient groups were meeting for the first time. Nonetheless, all sessions were characterized by lively discussions regarding these elders’ experiences receiving care for LBP. While group members turned to the moderator with their answers early in the discussions, by midway through the sessions the participants spoke directly to one another, nodded or shook their heads in response, and offered supportive or countering opinions.

**Table 2:** Participant characteristics (N = 48)

**Characteristic**	
Age, mean (SD)	75.2 (8.0)
Female, %	77
Ethnicity, Hispanic %	4
Race, white %	96
Marital status, %	
Married or living with significant other	44
Widowed	40
Divorced or separated	15
Never married	2
Education, %	
Some grade school or high school	17
High school graduate	35
Some college or training program	29
College graduate	18
Employment, %	
Retired	85
Employed	15
Overall health, %	
Excellent to very good	27
Good	35
Fair	29
Poor	6
Overall pain in past 24 hours (0-10 NRS), mean (SD), range	4.7 (2.5), range 0-9
Back pain in past 24 hours (0-10 NRS), mean (SD), range	4.2 (2.4), range 0-9
Healthcare providers seen for back pain, %	
Doctor of chiropractic	69
Medical doctor	60
Physical therapist	38
Doctor of osteopathy	17
Massage therapist	8

*SD* Standard deviation.

*NRS* Numerical Rating Scale.

### Back pain causes and consequences

Participants reported many LBP causes including traumatic injuries from motor vehicle crashes, war wounds, occupational injuries, lifting, pregnancy, domestic violence, or falls. Participants also identified anatomic or physiologic causes including pinched nerves, collapsed vertebrae, degenerative discs or spinal stenosis attributed to aging, heredity or poor posture. Conditions such as arthritis, knee or hip replacements, or diabetes often accompanied LBP. Onset varied with some participants reporting LBP for 30 to 50 years, while for others LBP coincided with retirement. Intensity ranged from an annoyance to debilitating. Most respondents functioned with LBP, but many modified ADLs to lessen or prevent pain. Participants were cautious during lifting, driving, vacuuming, mowing, recreation, and with sudden movements.

### Healthcare seeking experiences

When these older adults experienced LBP, they attempted to self-manage the pain with over-the-counter medications (e.g., acetaminophen, ibuprofen, aspirin, naproxen, creams), heat or cold applications, position changes, and self-massage. Exercise sometimes triggered LBP, but also was a preventive or relieving strategy and included walking, swimming, stretching and yoga. Participants sought professional care when LBP became burdensome with pain relief as the primary motivator (Table 3). These older adults chose treatment from two types of primary care providers: family or internal medicine physicians and doctors of chiropractic. Participants also received care from physical therapists, neurologists, massage therapists, and orthopedists. Most participants avoided injections or surgery stating they would rather “live with pain.”

**Table 3:** Expectations for medical and chiropractic treatment of low back pain

	**MD clinic (n = 8)**		**DC clinic (n = 6)**		**Senior center (n = 12)**			**Senior housing (n = 22)**		
**Medical treatments for LBP**	**M**	**M**	**D1**	**D**	**C**	**C**	**C**	**H**	**H**	**H**
	**1**	**2**	**1**	**2**	**1**	**2**	**3**	**1**	**2**	**3**
Pain relief expected	✓	✓	✓		✓	✓	✓	✓	✓	✓
Prescription medicine suggested	✓	✓	✓	✓	✓	✓	✓	✓	✓	✓
X-ray or diagnostic tests performed	✓	✓	✓	✓	✓	✓	✓	✓	✓	✓
Self-care/exercise recommendations	✓	✓	✓	✓	✓	✓	✓		✓	✓
Referral to specialist or pain clinic	✓		✓	✓	✓	✓	✓	✓	✓	✓
Surgery or injection recommendation	✓				✓	✓	✓	✓	✓	✓
Referral to doctor of chiropractic	✓	✓		✓			✓	✓	✓	
**Chiropractic treatments for LBP**	**M**	**M**	**D**	**D**	**C**	**C**	**C**	**H**	**H**	**H**
	**1**	**2**	**1**	**2**	**1**	**2**	**3**	**1**	**2**	**3**
Pain relief expected	✓	✓	✓	✓		✓	✓	✓	✓	✓
Hands-on treatment/adjustment given	✓	✓	✓	✓		✓	✓	✓	✓	✓
Treatment of the cause of LBP problem	✓	✓	✓	✓		✓	✓	✓	✓	✓
Loosen up joints/maintain mobility	✓	✓	✓	✓		✓	✓	✓		
Other modalities (TENS, instruments)	✓	✓	✓	✓	✓		✓	✓	✓	✓
X-ray or diagnostic tests performed				✓		✓	✓	✓	✓	✓
Chiropractic maintenance care helps	✓	✓	✓	✓		✓	✓	✓	✓	✓
Adjustment provides short-term relief	✓	✓		✓		✓	✓	✓	✓	
Treatment approaches vary by DC	✓	✓	✓			✓		✓	✓	✓
Referral to medical doctor	✓	✓						✓	✓	✓
Self-care/exercise recommendations		✓		✓		✓	✓			

MD = Medical Doctor.

DC = Doctor of Chiropractic.

LBP = Low back pain.

M# = Medical clinic focus group 1 or 2.

D# = Chiropractic clinic focus group 1 or 2.

C# = Senior center focus group 1, 2 or 3.

H# = Senior housing focus group 1, 2 or 3.

TENS = Transcutaneous electrical nerve stimulation.

The consensus across groups was that older adults who saw a medical doctor for LBP might receive a prescription, self-care recommendations, or referral to specialists or physical therapy (Table 3). As one SC participant said: “Usually they’ll take some x-rays to see what’s going on. Then what they probably do is give you some pills to mask the pain.” Others felt medical care addressed her back problems very well: “Last one I had when my back hurt, they gave me some medicine…it got rid of it [the pain] right now.”

Participants across groups considered chiropractic a primary, not complementary, LBP treatment. Participants said DCs offered many modalities, but expected chiropractors to provide “hands-on” treatments or spinal manipulation to deal with the cause of the pain (Table 3). One participant noted, “You expect them to give you adjustments to…not only to loosen you up, but maybe take the pain out of your spine or out of your joints. A regular doctor will not give you an adjustment. He can only give you medication.” Others, like this SC member, reported DCs also talked with them at length about their condition: “Chiropractors, they spend time with you, discussing what’s going on and they don’t rush you through.”

### LBP Co-management facilitators

When presented with the idea, most participants believed collaboration by a MD-DC team could be a positive treatment approach to LBP (Table 4). Participants noted DCs and MDs did sometimes refer patients: “My chiropractor told me go to the medical doctor to make sure there’s nothing else going on.” While many participants sought LBP treatment from multiple providers, none had received concurrent care from a MD and DC who communicated about their diagnosis and plan of care. As one SC participant said, “I like the idea that they work together, that they communicate, that they even discuss what the plan should be.” Several elders, including this SH participant, questioned why co-management between these providers was not a current standard of practice: *“*They consult with other medical doctors, so if they consulted with your chiropractor too, in the same context, I think it would benefit everybody in the long run.”

**Table 4:** Older adult perceptions of potential facilitators and barriers of low back pain co-management by MDs and DCs

	**MD clinic**		**DC clinic**		**Senior center**			**Senior housing**		
**LBP Co-management facilitators**	**M**	**M**	**D**	**D**	**C**	**C**	**C**	**H**	**H**	**H**
	**1**	**2**	**1**	**2**	**1**	**2**	**3**	**1**	**2**	**3**
Co-management would benefit LBP patient	✓	✓	✓	✓	✓	✓	✓		✓	✓
Collegial approach needed for LBP care	✓	✓	✓	✓	✓	✓	✓	✓	✓	✓
Historically strained relations between MDs and DCs has improved over time	✓	✓	✓	✓		✓	✓	✓	✓	✓
Referrals/consultations/phone calls between MD and DC needed for co-management	✓	✓	✓	✓		✓	✓	✓	✓	✓
Coordinated LBP treatment plan	✓	✓	✓	✓	✓	✓	✓		✓	✓
Health record/X-ray sharing required	✓	✓		✓	✓	✓	✓	✓	✓	✓
Offices located together or nearby		✓	✓		✓		✓	✓		
Individualized care for LBP	✓	✓				✓	✓	✓	✓	
**LBP Co-management barriers/concerns**	**M**	**M**	**D**	**D**	**C**	**C**	**C**	**H**	**H**	**H**
	**1**	**2**	**1**	**2**	**1**	**2**	**3**	**1**	**2**	**3**
Medication: do not want to use any medicine, side effects, no improvement, narcotic addiction, masking pain	✓	✓	✓	✓	✓	✓	✓	✓	✓	✓
Financial costs or insurance issues from receiving care from 2 or more doctors	✓	✓	✓		✓	✓	✓	✓	✓	✓
Providers may not support a co-management approach to LBP care	✓	✓	✓		✓	✓	✓	✓	✓	✓
Chiropractic: Side effects, providers who do not treat a condition, no improvement	✓	✓	✓			✓	✓	✓	✓	✓
Receiving care from multiple doctors	✓				✓	✓	✓	✓	✓	✓
Duplicate/unneeded tests/treatments	✓				✓	✓	✓	✓	✓	
Conflicting information or treatments	✓				✓	✓	✓	✓	✓	
Scheduling and transportation concerns					✓	✓	✓	✓	✓	✓
Questionable benefit of either medical, chiropractic, or co-management based on previous LBP treatment experience					✓	✓	✓	✓	✓	✓

MD = Medical Doctor.

DC = Doctor of Chiropractic.

LBP = Low back pain.

M# = Medical clinic focus group 1 or 2.

D# = Chiropractic clinic focus group 1 or 2.

C# = Senior center focus group 1, 2 or 3.

H# = Senior housing focus group 1, 2 or 3.

Participants emphasized that in a co-management situation doctors should respect one another as colleagues. Most focus groups had a perception of strained professional relationships between MDs and DCs in the past, but many felt this situation has changed for the better over their lifetimes. Many participants also stated the doctors should work physically close to one another for optimal collaboration: “If they had an office together, and would discuss the plan, it would be a good thing for me, because one is doing this [chiropractic], and one is medicine.”

One SH participant suggested collaborating doctors should at least communicate by telephone: “I wouldn’t mind if they talked to each other on the phone and discussed my case.” Participants also recommended health record sharing. Few had privacy concerns for such interdisciplinary communication, especially if the burden of completing multiple forms or transferring records was minimized. Another SH participant said: “If they shared the medical chart that they have on you with the chiropractor, with the charts he has on you, between the two of them they could resolve a lot of things without the medication if they compared notes.” A medical patient concurred: “I think information sharing is the main thing.”

### LBP Co-management concerns

While generally agreeing that MD-DC collaboration was a good idea, some participants wondered about its benefit or efficacy, particularly when dissatisfied from previous experiences receiving care from either provider (Table 4). Some participants were concerned about overlap in testing or treatment. As a medical clinic participant said, “You go to a medical doctor and he takes x-rays or MRIs…and then you go to a chiropractor and he takes x-rays. I think you can overdo that process…but if they worked together and used the same tests… that helps.”

Most participants were concerned about prescription medicine use in any collaborative model. Many older adults, like this SH participant, reported they did not use their pain medicine: “I don’t take half the prescriptions. I tear them up…I won’t try it.” Some feared addiction and only took medicine, especially opioids, when the pain became “unbearable.” Still others reported medication side effects: “I could not take it, because it was too strong, and I was drowsy all the time.” Similarly, some participants noted chiropractic adjustments did not relieve their LBP for several treatments, provided short-term relief or produced side effects, such as muscle soreness.

The logistical aspects of LBP collaborative care also were concerns. Participants stated they could not afford the financial expense, including insurance co-pays and out-of-pocket costs, associated with seeing different specialists for multiple co-morbidities. One SH participant said: “They could send me to all the specialists they want, but I don’t have the money to pay for it.” Older adults who had mobility issues, used public transportation, or lived in senior housing would need special travel arrangements. This SH participant said, “The time involved… you go to one doctor and then you’re going to another one that’s across town…my friend’s taken me to all of my appointments.” Scheduling was problematic, as a SC participant noted: “My biggest problem is getting through the office secretary for a time. ‘We can see you in four, five days. Otherwise go to the emergency room.’ What good is that, because they will say, ‘Well, go to your family doctor.’ You get the runaround.*”*

Participants also thought some providers might not engage in collaborative practice, even if the patient was interested. As a SC participant said, “I would not like my physician to say, ‘Do not seek chiropractic treatment.’ And I would not like my chiro to say, ‘You don’t need any other help. I can do everything for you.’ I would like them to work together*.*”

### Patient-centered communication

Patient-centered communication was essential for LBP collaborative care, or for *any* interaction between a patient and healthcare provider (Table 5). Participants across groups reported professionals from many disciplines responded to LBP concerns with statements such as, “You’re fine…for your age.” Others were frustrated when providers prioritized other health conditions over LBP: “I go to [doctor] and his only concern is my diabetes. I tried to talk to him about back pain…It’s ten minutes in and you’re gone, which is too bad.”

**Table 5:** Older adults’ recommendations regarding patient-centered communication for LBP co-management by MDs and DCs

	**MD clinic**		**DC clinic**		**Senior center**			**Senior housing**		
	**M**	**M**	**D**	**D**	**C**	**C**	**C**	**H**	**H**	**H**
	**1**	**2**	**1**	**2**	**1**	**2**	**3**	**1**	**2**	**3**
Providers should use respectful, honest communication between each other and with patients		✓	✓	✓	✓	✓	✓	✓	✓	✓
Provider must listen to the patient	✓	✓		✓	✓	✓	✓	✓	✓	✓
Patient must let doctors know they are seeing other providers for LBP	✓	✓	✓	✓	✓	✓	✓	✓	✓	
Providers should explain condition, diagnosis, treatment and prognosis		✓	✓		✓	✓	✓	✓	✓	✓
Patients should use their own judgment about any advice for LBP		✓	✓	✓		✓		✓	✓	✓
Providers should spend more time with patient				✓	✓	✓	✓	✓	✓	✓
Providers should not blame LBP on the older adults’ health condition or age		✓	✓			✓	✓	✓	✓	✓
Providers should discuss pain openly with their patients					✓	✓	✓	✓	✓	✓
MD-DC should resolve disagreements and present unified LBP approach to patient	✓				✓	✓	✓	✓		
If dissatisfied with care, patient should change doctors or get another opinion	✓	✓	✓			✓		✓	✓	✓

MD = Medical Doctor.

DC = Doctor of Chiropractic.

LBP = Low back pain.

M# = Medical clinic focus group 1 or 2.

D# = Chiropractic clinic focus group 1 or 2.

C# = Senior center focus group 1, 2 or 3.

H# = Senior housing focus group 1, 2 or 3.

Some older adults, like this SC participant, felt doctors treated them as a number rather than as a person: “There’s few doctors nowadays that actually sit and listen to you, and haven’t made up their mind what they’re to do for you ahead of time.” Participants preferred providers who treated them with respect, cared for their individual needs, and recognized the patient as the expert. As a SC participant stated: “I want respect shown to the patient. There isn’t anybody that knows their body any better than that person and for a doctor to, or a nurse or anyone to say, ‘You shouldn’t be having that kind of pain’…You need to teach, treat each separately.”

Some older adults felt providers did not offer enough information about their LBP. Most, like this SC participant, wanted honest communication regarding their LBP condition, including its diagnosis, prognosis and treatment: “When you tell them you have an issue, things could be changing, but they just say, ‘Well, your old back is hurting you. Let’s try this medicine.’ I would like to know what changes are going on, and what’s going on, and why.” These older adults also thought patients should be accountable when talking about their treatment from other providers. One SH participant said, “I think you definitely have to because that way it’s a group effort to get you better.” Others, like this SC participant, noted the importance of such transparency on health history forms: “They knew I was doing that because you fill out forms when you go in the office, ‘Are you being seen by…’ Yes, yes, yes, yes, so they already know what I was doing.”

Participants across groups proffered that collaborating doctors should provide consistent recommendations. As this SC participant noted: “I was just thinking you go to your chiro and he tells you one thing. You go to your doctor and he says, ‘Oh, I don’t think you should be doing that.’ What if they worked together? I think that would be really good.” When asked how to reconcile divergent recommendations, many participants said disagreements about the plan of care demonstrated the providers were not working together. Another participant questioned providers who put the patient in the middle with conflicting recommendations: “They’re not communicating with each other…Right? Wouldn’t you say that? If they’re going to do the complete opposite, then they don’t really have any respect for each other. They don’t talk about it. How are we supposed to determine who’s right, who’s wrong?”

Some participants reported that they would resolve this issue by seeking a third opinion, while others stated a disagreement might make them reconsider care from either doctor: “I probably wouldn’t go to either one.” Most participants would consider both recommendations and use their own judgment to decide on the best approach for their LBP. One SH resident said, “I would evaluate both situations…find something that I could read up on…and make my own decision as to which I’d follow up because I know my body better than any doctor.” Another SH participant summed up the benefits of patient-centered communication within the context of MD-DC collaboration: “I would expect them to get to the base of the problem. I think the biggest thing…tell the patient what’s going on and what’s going through the medical person’s mind and…the chiropractor, explain it…that makes a big difference.”

## Discussion

While previous research has evaluated collaborative models for primary care [43,55,56], this study is among the first to assess older adults’ preferences for LBP co-management by MDs and DCs in primary care settings. The dynamics of collaboration between healthcare providers may vary widely, as identified by Boon and colleagues [57,58], who have developed a seven model conceptual framework of team-oriented health care delivery. In this study, our participants largely described models of LBP treatment that may be best described as parallel practice with some consultative practice [58]. For example, most participants sought pain relief from both MDs and DCs, but no participant had experienced MD-DC collaborative care for LBP in which their providers shared health information or coordinated their care in any way. Nonetheless, our participants considered MD-DC collaboration a feasible treatment model. These older adults emphasized honest and respectful communication between health professionals for LBP co-management success. Participants also stressed the importance of providers’ direct, honest, and consistent communication with older adults suggesting any co-management model must embrace a patient-centered care approach, a finding that echoes other studies of patient preferences for doctor-patient communication [31,43,44,59-61].

Older adults were interested in talking with doctors about their diagnoses, prognosis and treatment options, as other research in primary care settings has shown [18,61,62]. A recent study found the quality of communication between LBP patients and 3 professional subgroups (MD, DC, and physical therapy) were worse the longer the patient had LBP and in older patients [63]. Physicians demonstrate low confidence and knowledge scores toward evaluation and treatment of LBP in elderly people [64]. While comparative studies for DCs are unavailable, chiropractic students are less knowledgeable about primary care, other than musculoskeletal conditions, than medical students [65]. Our findings suggest a MD-DC team approach may fill the knowledge and skill gaps of each provider, as primary care patients desire competent clinicians who know when to seek assistance from other professionals [66]. Intensive training and practice in communication skills may benefit health professionals who work with older adults and patients using CAM [61,67,68]. Healthcare providers should develop communication skills that assist patients with LBP to set realistic expectations about their treatment options, understand their potential outcomes, and engage in an active role in their therapeutic process [69].

Barriers to LBP co-management identified by these participants included financial costs, scheduling and transportation issues, and side effects from medication or chiropractic treatment, similar to other studies [42,43,61]. Participants thought some providers would be unwilling to engage in collaborative practice, a finding noted in other studies that have explored combining conventional and complementary approaches to healthcare [42,51]. Participants viewed information sharing between doctors through provider referral, communication by telephone or in shared practice settings, or via computer-mediated health record exchange as an essential facilitator of LBP co-management. Previous studies of interdisciplinary practice by MDs and DCs revealed little concurrent care, referral or record sharing by these providers [23,27-30] suggesting the need for pragmatic co-management models to guide such practice innovations.

Through this focus group study, we improved our model of collaborative care for older adults with back pain and the interprofessional context in which this model was tested [48]. Our results also have implications for clinical practice. These participants were uneasy about pain medicine because of potential side effects, a finding expressed by other primary care and CAM patients in other studies [18,43,70]. Some participants refused to take pain medicine and chose to “live with pain”. One preliminary study demonstrated structural brain changes in older adults with chronic LBP [71], suggesting elders’ decisions to “live with pain” may be detrimental not only to their quality of life [4,7,9] but their cognitive status as well [71]. Health providers who co-manage LBP in older adults should be aware of their concerns regarding medication safety, the preferences of many elders for minimal medication use for pain relief, and the need to ascertain whether their patients are using their medications as prescribed.

### Study limitations

This study has limitations. While focus group methodology was used to elicit a range of opinions [49] about LBP collaborative care, participants may not have shared their thoughts or feelings about this topic completely. Selection bias may be present. Our sample may differ from the target population of older adults with back pain given the unique geographic setting of our study. Palmer College of Chiropractic was established as the founding chiropractic college in 1897; subsequently, community awareness of chiropractic as a treatment for LBP is very long-standing. While we are not aware of such statistics, it is reasonable to suggest that more people in the Quad-Cities would have tried both medical and chiropractic care for LBP than would be found in the general population. Thus, the participants in this study may be more open to LBP co-management approaches by MDs and DCs than other LBP patients. We recruited participants from a variety of settings; however, many older adults in this community did not have the opportunity to participate. Older adults who had not sought care from these clinics, resided in long-term care settings or were homebound, did not speak English or attend senior centers, and those closed to co-management were not represented. Several potential participants stated they were not interested in discussing back *pain*, as they sought to live fully despite occasional discomforts. These seniors suggested future recruitment efforts promote back *health*. Lastly, our study focused on older adults with LBP. While our findings also may apply to younger and middle-aged adults seeking care for back pain, the unique needs of those working-aged individuals may warrant additional study.

## Conclusions

This focus group study demonstrated an interest among older adults with back pain in co-management models by medical doctors and doctors of chiropractic. Older adults viewed collaboration between these providers as a potentially advantageous approach for back problems. Participants thought the combination of medical treatments and chiropractic adjustments might best achieve their goals for pain relief for low back conditions and improved physical function.

Older adults identified financial costs, scheduling and transportation issues, side effects from medication or chiropractic treatment, duplicate testing, and providers’ willingness to engage in collaborative care models as potential barriers to low back pain co-management. Facilitators of back pain treatment provided jointly by medical doctors and doctors of chiropractic included a collegial approach between providers, referral and consultation relationships, health record sharing, and co-located clinics. Patient-centered communication that included respect for the older adult as the expert in his or her own body, an openness to discussing chronic pain and diagnoses, refraining from blaming back pain on the patient’s age, and an individualized approach to treatment were considered key components of LBP collaborative care.

## Abbreviations

ADL: Activities of daily living; CAM: Complementary and alternative medicine; DC: Doctor of chiropractic; LBP: Low back pain; MD: Medical doctor; NRS: Numerical rating scale; SC: Senior center; SD: Standard deviation; SH: Senior housing; TENS: Transcutaneous electrical nerve stimulation.

## Competing interests

The authors declare they have no competing interests.

## Authors’ contributions

The study was conceived and the interview designed by CMG, KJL, SAS, MAH, MEJ, and AAA. SAS, MEJ, AAA, and CMG were involved in participant recruitment. The focus group moderating and data analysis team included KJL, SAS, and MAH. KJL and SAS drafted the manuscript. MAH, CMG, MEJ, and AAA provided manuscript revisions. All authors read and approved the final manuscript.

## Pre-publication history

The pre-publication history for this paper can be accessed here:

http://www.biomedcentral.com/1472-6882/13/225/prepub
